# Acromegaly: a Latin American perspective

**DOI:** 10.1007/s11102-013-0518-9

**Published:** 2013-09-19

**Authors:** Moisés Mercado

**Affiliations:** 1 Faculty of Medicine, Universidad Nacional Autónoma de México, Mexico City, Mexico; 2 Endocrine Service, and Experimental Endocrinology Unit, Hospital de Especialidades, Centro Médico Nacional Siglo XXI, Instituto Mexicano del Segero Social, Aristóteles 68, Polanco, 11560 Mexico City, Mexico

Clinical endocrinology can be viewed as a polarized discipline. On one end of the spectrum, endocrinologists look after patients with very common disorders such as type 2 diabetes, which constitute global health care problems of pandemic proportions. On the other end, the specialty deals with very rare conditions, frequently regarded as “exotic”. Acromegaly is one of these low-prevalence conditions, yet it has traditionally been the focus of attention of eminent characters in the history of medicine, from neurologists like Marie [1], to neurosurgeons like Cushing [2], to physiologists like Houssay [3]. Perhaps the reason behind such an emphatic interest in this condition is that as rare as it can be, acromegaly can be viewed as an in vivo model that helps us understand a myriad of biological processes. Etiologically, the molecular mechanisms responsible for the development of GH-secreting pituitary adenomas-the ultimate cause of acromegaly/gigantism in over 98 % of cases-constitute oncogenic models that elegantly link abnormalities in cell proliferation with autonomous hormonal hypersecretion [4, 5]. Pathophysiologically, understanding the systemic consequences of the GH/IGF-1 excess has generated crucial knowledge in regards to issues such as glucose metabolism, insulin resistance, vascular endothelium physiology, hypertension, renal sodium handling and even neoplasia formation [6]. Clinically, in order to adequately care for the acromegaly patient, one has to be a well-rounded internist, capable of diagnosing and treating all the complications and co-morbidities of the disease [6]. Not less relevant is the fact that the proper management of acromegaly should include the participation of a wide range of specialists, from skilled pituitary neurosurgeons and radiation oncologists, to otolaryngologists, maxillo-facial surgeons and even psychiatrists or clinical psychologists. Undoubtedly, this multidisciplinary team has to be coordinated by an endocrinologist with significant experience in pituitary disorders.

Since 1999, an international Acromegaly Consensus Group, sponsored by the Pituitary Society and the European Neuroendocrinological Association, has met periodically to evaluate the different aspects of acromegaly management. As a result of these gatherings, several reports have been published issuing guidelines and recommendations [7–12]. Although the publications derived from the early consensus meetings do not follow a systematic, evidence-based methodology [7–9], the last three have used the GRADE system to rank their recommendations [10–12].

The care for patients with complex diseases such as acromegaly varies between different regions of the World due to specific local circumstances such as the availability of qualified specialists and economic resources to pay for costly pharmacological treatments and radiotherapeutical interventions. In recognition of these regional variations, the Acromegaly Consensus Group has included the participation of specialists from Brazil and Mexico. Latin America cannot be considered a uniformly underdeveloped region of the World. Although health care priorities are somewhat different from those that prevail in Europe or the United States, most Latin-American Countries have tertiary care, referral centers fully capable of caring for the patient with acromegaly. In most instances, we find government-based subsidized health care co-existing with private medicine, and patients with acromegaly receive multimodal care at referral centers, just like in Europe and the United States. In this regard, perhaps the main difference is that in Latin America, medical facilities with state of the art technology and appropriate personnel (including pituitary neurosurgeons) are centralized in two or three large cities. Thus, in Latin America, a patient diagnosed with acromegaly has to be canalized to these referral centers [13]. In most Latin American countries the cost of pharmacological treatment, particularly, somatostatin analogs is absorbed by the government. Both octreotide and lanreotide depot formulations are actively commercialized and prescribed at least in Argentina, Brazil, Chile, Colombia, Mexico, Peru and Venezuela. The GH receptor antagonist pegvisomant, on the other hand is utilized less frequently due to its higher cost. Radiation therapy in most of its modalities is probably used more frequently in the region, because of its relatively low cost and proven efficacy. Many of these Latin American centers are university hospitals actively engaged in clinical research. With increasing frequency, multidisciplinary groups from these centers publish their experience in the diagnosis and management of acromegaly. Several important Latin American publications have appeared in the medical literature, covering the oncogenesis of somatotrophinomas [14, 15], the clinical aspects of acromegaly [16] and its complications [17, 18], as well as the results of the surgical [19, 20], pharmacological [21–24] and radiotherapeutical management of the disease [25]. Interestingly, the outcomes of transsphenoidal surgery, radiotherapy and therapy with somatostatin analogs described in these publications are comparable to those reached in academic institutions from Europe and the United States [19–25].

Many Latin American specialists working in the field of neuroendocrinology share the idea that an acromegaly consensus adapted to the social, cultural, political and economic realities of the region is indispensable. Such a consensus meeting took place for the first time in Mexico City in 2007 [26]. In October 2012 a group of specialists from Argentina, Brazil, Colombia, Mexico and Venezuela gathered in Oxford, United Kingdom to review the current recommendations for the diagnosis, treatment and follow up of acromegaly, as they pertain or apply to the complexities of medical practice in Latin America. A case-based approach was chosen to better illustrate the different aspects of acromegaly care. Prior to the meeting, specialists from the region were invited to submit case presentations regarding early diagnosis, clinical presentation, identification and treatment of co-morbidities, the role and hierarchy of the different treatment modalities as well as the appropriate follow up of the disease. A steering committee selected some of these cases for presentation and discussion during the meeting. In this supplement of Pituitary we present the most representative of these cases and analyze the aspect of acromegaly care they allude to. We sincerely hope that this material will help readers not only from Latin America, but worldwide overcome the diagnostic and therapeutic dilemmas frequently encountered in the management of this condition.
